# Supplementary material for: Liter-scale manufacturing of shelf-stable plasmid DNA/PEI transfection particles for viral vector production
Source: Mol Ther Methods Clin Dev. 2024 Jan 22;32(1):101194. doi: 10.1016/j.omtm.2024.101194 (PMC10863326; doi:10.1016/j.omtm.2024.101194)
Supplement: Document S1. Supplemental methods and Figures S1 and S2 [file mmc1.pdf]

OMTM, Volume *32*

Supplemental information

Liter-scale manufacturing of shelf-stable plasmid DNA/PEI transfection particles for viral vector production

Yizong Hu, Brendan A. Eder, Jinghan Lin, Sixuan Li, Yining Zhu, Tza-Huei Wang, Ting Guo, and Hai-Quan Mao

Supplemental Methods

The fluorescence deconvolution algorithm has been used to analyze the payload in the nanoparticles. In our previous work, the DNA content in DNA/PEI and DNA/PEI-g-PEG nanoparticles (*Nano Letters* 2014, 14 4729-4735) and the mRNA content in lipid nanoparticles (*Nature Communications* 2022, 13, 5561) were analyzed by this technique. The same method was applied to analyze the pDNA payload in the size-controlled pDNA/PEI nanoparticles described in this report. For the nanoparticles with a measured diameter of 50 nm, 150 nm and 200 nm by dynamic light scattering (DLS), 100% (w/w) of the pDNAs were Cy5 labeled; Nanoparticles with 300 nm and 400 nm were loaded with 2% Cy5-pDNA.

For the 100% labeled condition, the pDNA payload was calculated as follows: First the fluorescence burst size distribution of the single molecule pDNA-Cy5 (D_{DNA}) and 100% Cy5 labeled pDNA/PEI nanoparticles (D_{NP}) were obtained by CICS, quantified by single particle fluorescence analysis, and D_{DNA} is normalized by its number of events. Due to the multiplicative nature of the single particle fluorescence, the distribution of the fluorescence is better described by a lognormal than normal distribution, thus quantified by logarithmic binning. The distribution of the single molecule pDNA-Cy5 (D_{DNA}) was used as a basis and a set of basis distribution $D_{DNA,n}\big|_{n=1,2,\ldots,N}$ were generated by multiplying D_{DNA} by a scaling factor n. For each basis distribution, $D_{DNA,n}$, the sum up of the distribution with all the bins is unity. I_B is the number of bins for the distribution:

$$\sum_{i=1}^{I_B} D_{DNA,n}(i) = 1\Bigg|_{n=1,2,\ldots,N}$$

$D_{DNA,n}(i)$ represents the proportion of the distribution in i-th bin of the basis of n pDNAs. I_B is the number of bins for the distribution. The maximum of n, N, is chosen to be a factor (k, typical around 4-6 times) of the ratio of the geometric mean of D_{NP} ($\mu_{D_{NP}}$) to D_{pDNA} ($\mu_{D_{DNA}}$) to ensure N is large enough to cover the whole payload range.

$$N = k\frac{\mu_{D_{NP}}}{\mu_{D_{DNA}}}$$

An estimated pDNA/PEI nanoparticle distribution (${D_{NP}}^*$) was constructed by summing up all the generated basis distributions of n pDNAs multiplied by a weight (w_n) representing the estimation of the number of nanoparticles encapsulating n pDNAs. The w_n is a non-negative integer that is no larger than the total number of pDNA/PEI nanoparticle events (N_{NP}).

$${D_{NP}}^* = \sum_{n=1}^{N} w_n \times D_{DNA,n}$$

$$w_n \in [0, N_{NP}], n = 1,2, \ldots, N$$

The fitted nanoparticle distribution ($D_{NP}{}^*$) was compared to that of the true distribution (D_{NP}) by calculating the difference based on each bin in the distribution. For each bin, we have the estimated number of the events in each bin given by

$$N(i)^* = D_{NP}(i)^* = \sum_{n=1}^{N} w_n \times D_{DNA,n}(i)$$

Then the total number of estimated events N^* is

$$N^* = \sum_{i=1}^{I_B} N(i)^* = \sum_{i=1}^{I_B} \sum_{n=1}^{N} w_n \times D_{DNA,n}(i) = \sum_{n=1}^{N} w_n$$

The chi square (χ^2) is calculated as the difference between the estimation and the true distribution, plus the difference between the estimated total number of events and the true value, as shown below:

$$\chi^2 = \sum_{i=1}^{I_b} \frac{(N(i) - N(i)^*)^2}{N(i)} + \alpha(N_{NP} - N^*)^2$$

A penalty factor (α) was chosen to be 0.1 to ensure N^* is < 1% off from N_{NP}.

The best fit was optimized by minimizing χ^2 using a simulated annealing algorithm in MATLAB, which gave the best estimate of the number of nanoparticles encapsulating the corresponding number of pDNAs per particle, w_n. The frequency f_n of the nanoparticles encapsulating n copies of pDNA was calculated as

$$f_n = \left.\frac{w_n}{N_{NP}}\right|_{n=1,2,\ldots,N}$$

The average payload of the whole population, μ, was calculated as

$$\mu = \sum_{n=1}^{N} n \times f_n$$

For the 100% pDNA-Cy5 labeled condition (i.e., 50 nm, 150 nm, and 200 nm nanoparticles), the payload distributions and average payload were obtained as described above.

Modifications were made to compensate for the nanoparticles with 2% pDNA-Cy5 labeling (i.e., 300 nm, and 400 nm nanoparticles). First, the payload distribution at 2% labeled condition, $f_{n,2\%}$, with payload number range from 1 to $N_{2\%}$, was calculated as described above. Since only 2% pDNAs were Cy5 labeled,

a significant number of nanoparticles may not have a fluorescent label. The number of the Cy5 labeled pDNAs in the particles, N, follows a binomial distribution, B:

$$P(N = x) \sim B(x, n, p)$$

Where n is the detected number of payload, p is the labeling ratio ($p = 0.02$).

The portion of the unlabeled nanoparticles was given by:

$$P(N = 0) = \sum_{n=1}^{N_{2\%}} B(0, n, p) \times f_{n,2\%}$$

An overestimation factor K_{oest} due to neglecting the portion of nanoparticle that encapsulated no Cy5-labeled pDNA (n = 0) in the payload quantification was calculated as:

$$K_{oest} = \frac{1}{1 - P(N = 0)}$$

And to include the nonfluorescent portion in the payload distribution, $f_{n,2\%}$ was corrected given by

$$f_{n,2\%,corr} = \frac{[P(N = 0), f_{n,2\%}]}{sum([P(N = 0), f_{n,2\%}])}$$

Next, to back calculate the complete payload distribution from the 2% labeled results, a matrix mapping the binomial distribution of the 100% to 2% probability, M was constructed with the entry in n-th row and m-th column:

$$M_{n,m} = B(x, n, p)\big|_{n=1,2,\ldots,N_{100\%},\ m=1,2,\ldots,N_{2\%}+1}$$

Where $N_{100\%}$ is the largest payload number in the complete payload distribution, and $N_{2\%}$ is the highest payload in the payload distribution at 2% labeled condition, $f_{n,2\%}$. The 100% probability was obtained by dividing the corrected 2% payload distribution by the binomial probability matrix:

$$f_{n,100\%} = f_{n,2\%,corr}/M$$

And the average payload of the whole population, μ, was calculated as

$$\mu = \frac{\sum_{n=1}^{N_{100\%}} n \times f_{n,100\%}}{K_{oest}}$$

Supplemental Figures

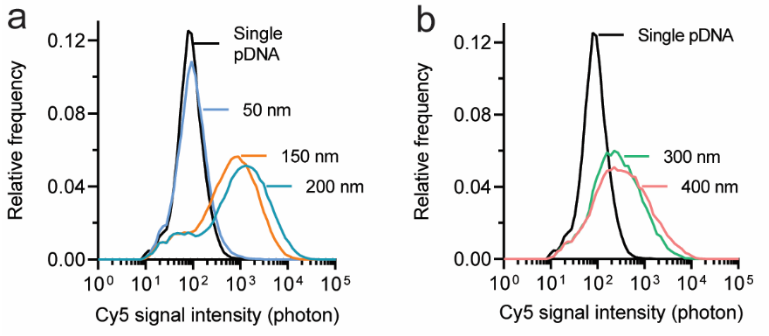

Figure S1. The distribution of Cy5 signal intensity from pDNA/PEI nanoparticle events detected by cylindrical illumination confocal spectroscopy (CICS) single-nanoparticle characterization technique.

(a) For pDNA/PEI nanoparticles loaded with 100% Cy5-pDNA at a size of 50, 150, and 200 nm, referencing to the signal intensity distribution of individual free Cy5-pDNAs; **(b)** For pDNA/PEI nanoparticles loaded with 2% Cy5-pDNA and 98% non-labeled pDNA at a size of 300 and 400 nm.

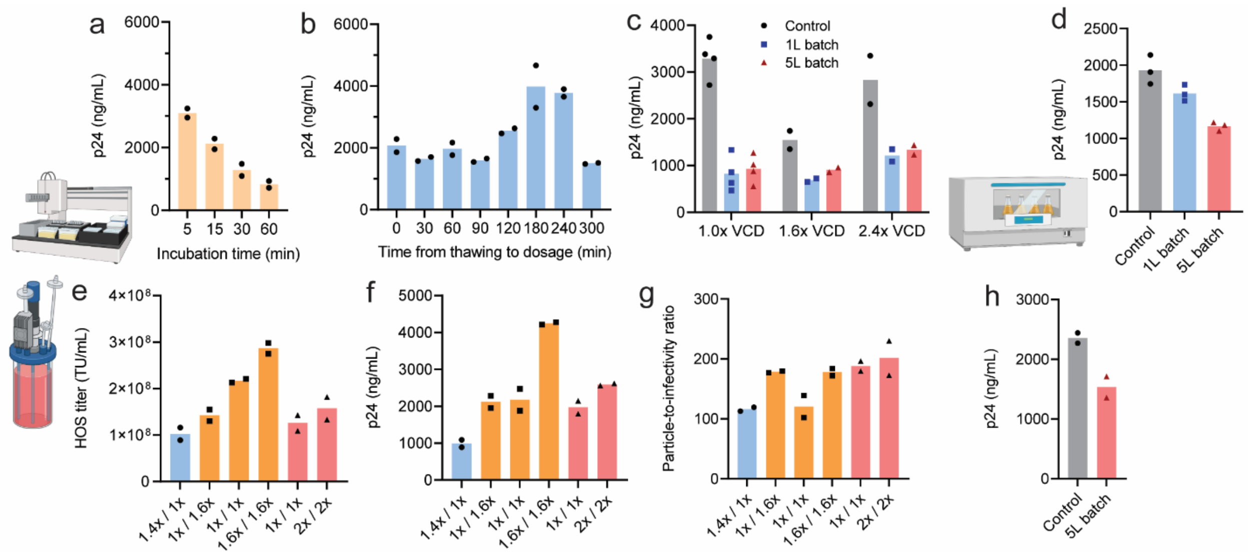

Figure S2. Additional data from lentiviral vector production using stable pDNA/PEI nanoparticles (NPs).

Additional data from lentiviral vector production is shown for **(a–c)** Ambr 15 culture system; **(d)** 100-mL shake flask system; and **(e–h)** 2-L in-house developed single-use bioreactors.

(a) p24 capsid protein quantifications from harvested LVVs produced by the conventional pDNA-PEI mixing method (control, main text **Figure 1a**), and the time on x axis indicates the time between mixing pDNA with PEI and dosing; **(b)** p24 capsid protein quantifications from harvest LVVs produced by stable NPs (the 1-L lot), and the time on x axis indicates the time between complete thawing and dosing under ambient temperature. **(c)** p24 capsid protein quantifications from harvested LVVs comparing the control with 1-L and 5-L lot NPs. 1x VCD represents the standard viable cell density at 24 h prior to NP dosing, while the dose was proportional to the VCD level. **(d)** p24 capsid protein quantifications from harvested LVVs comparing the control with 1-L and 5-L lot NPs in the 100-mL shake flask system with 1x VCD and 1x dose. **(e–g)** The 1-L lot NPs were used. The quantity and quality of LVVs produced in the experiments that varied dose and VCD are reported, and the x axis reads VCD level / NP dosing level. For example, 1.4x / 1x reads 1.4x VCD and 1x NP dose, in which 1x dose is the standard condition of 1 μg total pDNA/mL in culture. The experiments were carried out in three independent batches of bioreactor runs, and the columns with the same color (either blue, orange, or red) were from parallel experiments within the same batch of bioreactor run. **(h)** p24 capsid protein quantifications from harvested LVVs comparing the control with 5-L lot NPs in the 2-L bioreactor with 1x VCD and 1x dose.

Supplemental Videos

In all supplemental videos, the red color indicates Gal8-mRuby3, while the blue color indicates Cy5-tagged pDNA.

Video S1. The full recorded video of B16F10-Gal8-mRuby3 cells treated by 400-nm NPs, with all Z planes stacked to give a top-down view of a 3-D perspective.

Video S2. The full recorded video of B16F10-Gal8-mRuby3 cells treated by 50-nm NPs, at a single focal plane of Z = 10.

Video S3. The full recorded video of B16F10-Gal8-mRuby3 cells treated by 400-nm NPs, at a single focal plane of Z = 10.
