# Supplementary material for: Liter-scale manufacturing of shelf-stable plasmid DNA/PEI transfection particles for viral vector production
Source: Mol Ther Methods Clin Dev. 2024 Jan 22;32(1):101194. doi: 10.1016/j.omtm.2024.101194 (PMC10863326; doi:10.1016/j.omtm.2024.101194)
Supplement: Document S2. Article plus supplemental information [file mmc5.pdf]

Molecular Therapy
Methods & Clinical Development
Original Article

Liter-scale manufacturing of shelf-stable plasmid DNA/PEI transfection particles for viral vector production

Yizong Hu,[1,2,3,7] Brendan A. Eder,[4] Jinghan Lin,[1,3,5] Sixuan Li,[1,6] Yining Zhu,[1,2,3] Tza-Huei Wang,[1,2,6] Ting Guo,[4] and Hai-Quan Mao[1,2,3,5]

[1]Institute for NanoBioTechnology, Johns Hopkins University, Baltimore, MD 21218, USA; [2]Department of Biomedical Engineering, Johns Hopkins University School of Medicine, Baltimore, MD 21218, USA; [3]Translational Tissue Engineering Center, Johns Hopkins University School of Medicine, Baltimore, MD 21231, USA; [4]2seventy bio, Inc., Cambridge, MA 02142, USA; [5]Department of Materials Science and Engineering, Johns Hopkins University, Baltimore, MD 21218, USA; [6]Department of Mechanical Engineering, Johns Hopkins University, Baltimore, MD 21218, USA

The transfection efficiency and stability of the delivery vehicles of plasmid DNA (pDNA) are critical metrics to ensure high-quality and high-yield production of viral vectors. We previously identified that the optimal size of pDNA/poly(ethylenimine) (PEI) transfection particles is 400–500 nm and developed a bottom-up assembly method to construct stable 400-nm pDNA/PEI particles and benchmarked their transfection efficiency in producing lentiviral vectors (LVVs). Here, we report scale-up production protocols for such transfection particles. Using a two-inlet confined impinging jet (CIJ) mixer with a dual syringe pump set-up, we produced a 1-L batch at a flow rate of 100 mL/min, and further scaled up this process with a larger CIJ mixer and a dual peristaltic pump array, allowing for continuous production at a flow rate of 1 L/min without a lot size limit. We demonstrated the scalability of this process with a 5-L lot and validated the quality of these 400-nm transfection particles against the target product profile, including physical properties, shelf and on-bench stability, transfection efficiency, and LVV production yield in both 15-mL bench culture and 2-L bioreactor runs. These results confirm the potential of this particle assembly process as a scalable manufacturing platform for viral vector production.

INTRODUCTION

In vitro transfection of a production cell line, such as HEK293 cells, is widely used in the production of viral vectors.[1–3] It involves the intracellular delivery of plasmid DNA (pDNA) in a nanoparticle (NP) form, assembled by complexing with cationic polymers, such as PEIpro, a form of poly(ethylenimine) (PEI) developed by Polyplus-transfection, and suspended in a serum-reduced or serum-free culture medium in common laboratory and industrial practices.[4,5] Major challenges associated with this process include low stability of NPs in culture medium as NPs are added to cell culture over an extended period, rendering a tight time window to dose the mixture of pDNA and PEIpro into bioreactors (Figure 1A). Besides, each run to produce viral vectors requires a new pDNA/PEIpro batch to be made, jeopardizing lot-to-lot consistency in production yield and quality. These challenges amplify with scale-up efforts of transfection,[6,7] as neither complexing pDNA and PEIpro in liter volumes or liquid dispensing into bioreactors of hundreds of liters of volume could accommodate tight engineering and quality control (QC) metrics over the transfection particles, before and during the dosing process. In this regard, our aim was to manufacture shelf-stable and on-bench stable transfection particles with QC implemented based on the characteristics of pDNA/PEIpro NPs. By addressing the time-sensitive schedule of NP production and dosing time to cell culture, we expect to improve process reproducibility, lot-to-lot consistency, viral vector production efficiency, and eventually reduce the production cost of expensive viral vector-based gene therapies (Figure 1B).

In our previous work,[8] we identified that the size of pDNA/PEIpro NPs is a critical parameter in determining transfection efficiency in both adherent HEK293T cells and suspension HEK293F cells, and an optimal diameter resides near 400–500 nm, thus putting a conclusion to the previously observed dependence of transfection efficiency on incubation time and ionic strength of the mixture of pDNA and PEI.[9,10] Furthermore, we developed a kinetically controlled assembly method to produce stable pDNA/PEIpro NPs with an average size of 400 nm based on modulation of salt-induced charge screening and residual charge on NP surface at a lot size of <100 mL. The NPs showed high on-bench stability at ambient temperature for >24 h and >4-month shelf stability at −80°C. The process includes three mixing steps (Figure 1C), separated by flexible time windows that allow for reproducibility of NPs with tightly controlled size and consistent

Received 10 August 2023; accepted 18 January 2024;
https://doi.org/10.1016/j.omtm.2024.101194.

[7]Present address: Koch Institute for Integrative Cancer Research, Massachusetts Institute of Technology, Cambridge, MA 02139, USA

Correspondence: Ting Guo, 2seventy bio, Inc., Cambridge, MA 02142, USA.
E-mail: ting.guo@2seventybio.com
Correspondence: Hai-Quan Mao, Institute for NanoBioTechnology, Johns Hopkins University, Baltimore, MD 21218, USA.
E-mail: hmao@jhu.edu

This is an open access article under the CC BY license (http://creativecommons.org/licenses/by/4.0/).

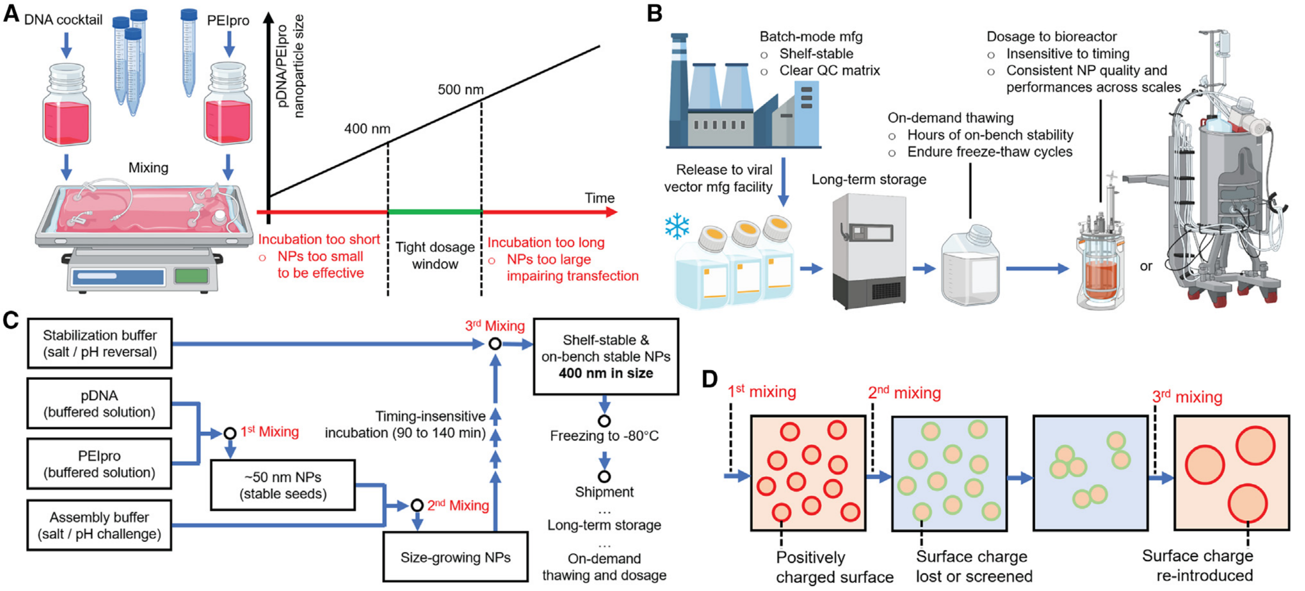

Figure 1. Process overview for scale-up production of 400-nm pDNA/PEIpro NPs

(A) The conventional and currently adopted methodology in industrial practices to prepare pDNA/PEIpro transfection particles immediately before dosing a cell culture. (B) A desired practice to produce viral vectors is depicted as manufacturing (mfg) of pDNA/PEIpro NPs with clearly defined QC matrix as a separate step. (C) The workflow of the scale-up production of 400-nm pDNA/PEIpro NPs involving three mixing steps that need implementation of scalable mixing techniques. (D) The NP size control mechanism. (A) and (B) were generated using BioRender.com.

transfection efficiency. Such highly stable NPs facilitate optimization of viral vector production process in bioreactors by removing variability associated with NP characteristics and transfection efficiency (Figure 1B). Nonetheless, fully validating the translational value of this method requires further scale-up to match the commercially relevant transfection and viral vector production lot scales. Therefore, generation of transfection vehicles at a lot size of liters and a DNA concentration of 50 μg/mL was set as a goal for this study.

Scalable mixing techniques have advanced significantly over the past decade. Representative mixing techniques include T-junction impinging jet mixing,[11] confined impinging jet (CIJ) mixing, which was used to produce the Pfizer-BioNTech COVID-19 vaccine,[12,13] paralleled microfluidic modalities,[14,15] etc. Description of the mixing dynamics and phase-separation kinetics during NP formation using CIJ mixers has been analyzed by Prud'homme et al. in 2003,[16] and it was used for flash nanoprecipitation in the preparation of drug-loaded polymer micellar NPs.[17,18] We adopted the CIJ mixing to produce polyelectrolyte complex NPs, such as pDNA/PEI,[19,20] protein/polymer complex NPs,[21–23] and pDNA/lipid[24] NPs through complexation-driven phase separation, i.e., flash nanocomplexation,[25] instead of solvent polarity-driven particle formation. Both techniques take advantage of the rapid and efficient mixing, thus achieving uniform phase separation, nucleation, aggregation, and particle growth.

In this report, we showcase the usefulness of CIJ mixing in realization of NP assembly to generate large NPs with diameters in hundreds of nanometers, particularly at larger lot sizes for industry adoption. Using a small CIJ mixer with a mixing chamber size of 27 μL and a dual syringe pump to establish continuous flow, 400-nm pDNA/PEIpro NPs were first produced at 1-L lot size. Next, a 5-L lot production was developed using a CIJ mixer with a 0.73-mL chamber volume, two solution reservoirs, and a pair of peristaltic pumps to provide a continuous and constant flow rate. QC analysis of the 400-nm pDNA/PEIpro NPs produced at both lot sizes confirmed that the size, uniformity, DNA content, transfection efficiency, and lentiviral vector (LVV) production yield in bioreactors at 2seventy bio, Inc., were consistent with that of NPs produced in small lot sizes. In the larger scale production method, the lot size is determined only by the size of the reagent reservoirs; therefore, this process can be easily adopted for larger scale manufacturing of pDNA/PEIpro transfection particles to address the challenges associated with lot-to-lot variability in viral vector production.

RESULTS

Characteristics of effective pDNA/PEIpro transfection vehicles

Our previously developed process[8] (summarized in Figure 1D) is based on a low-salt, low-pH solution environment upon 1st mixing of pDNA and PEIpro that ensures generation of colloidally stable ~50-nm NPs. The stability is conferred by charge repulsion between fully protonated PEI on NP surfaces at around pH 3.0. The 2nd mixing with assembly buffer brings the pH to 7.0 to deprotonate surface PEI, while a high salt concentration in this buffer screened the residual charge. With greatly reduced surface charge repulsion, the small NPs acting as building blocks grow steadily in size until the 3rd mixing step occurs when a stabilization buffer is added to reverse the solution

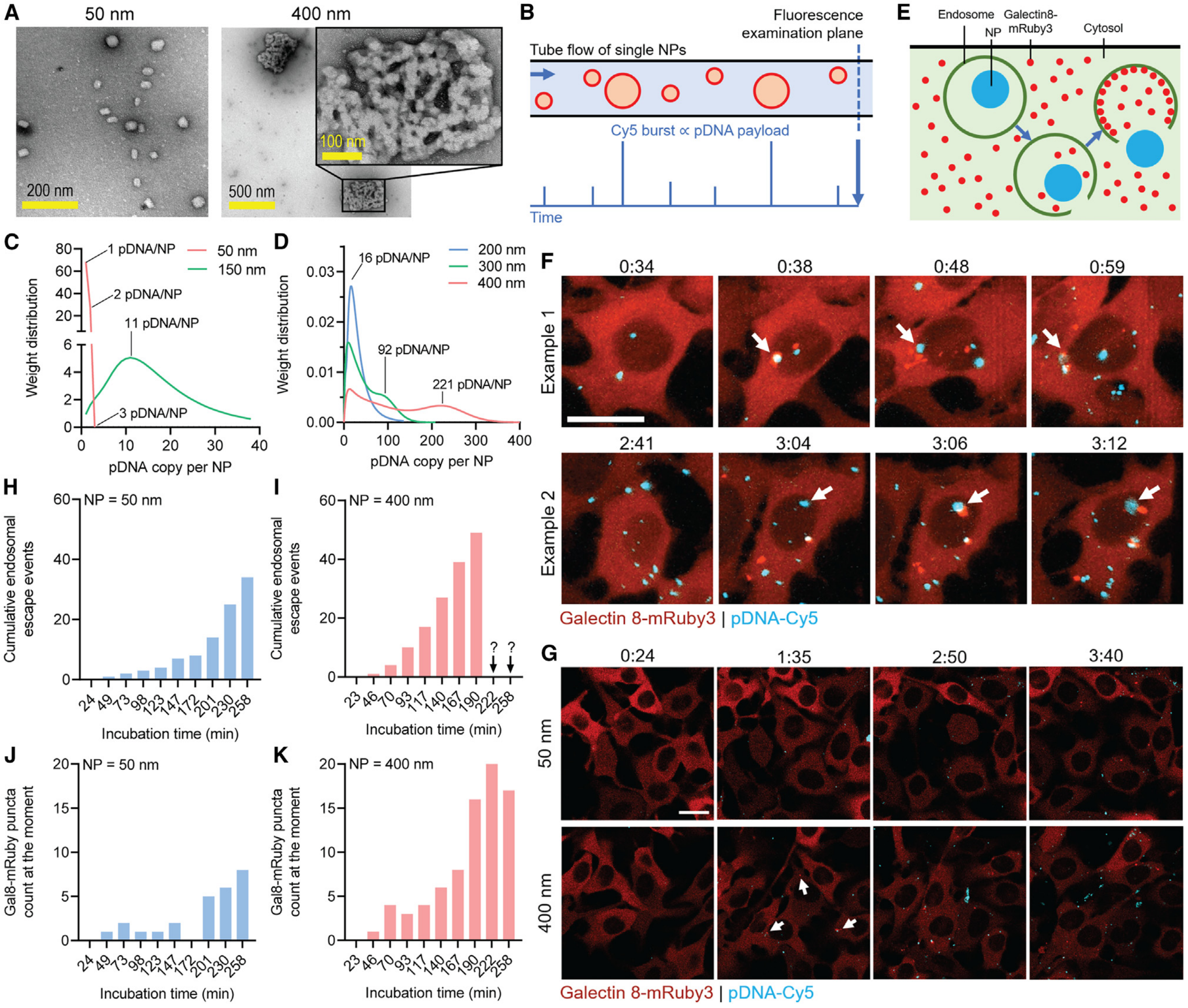

Figure 2. Effect of size of pDNA/PEIpro NPs on pDNA payload capacity and endosomal escape kinetics

(A) The TEM images of pDNA/PEIpro NPs at 50 nm (right after the 1st mixing step) and 400 nm (after the 3rd mixing step). (B) Characterization of pDNA payload at the single NP level using CICS. The weight distribution of pDNA payload of NPs at (C) 50- and 150-nm and (D) 200-, 300-, and 400-nm sizes. (E) Characterization of cellular uptake and endosomal escape of pDNA/PEIpro NPs using B16F10-Gal8-mRuby3 cell line. (F) Two representative endosomal escape events during live cell imaging on B16F10-Gal8-mRuby3 cells treated by 400-nm Cy5-pDNA/PEIpro NPs. The arrows point to the events of interest, in which cellular uptake (appearance of blue Cy5 spots), endosomal escape (appearance of red mRuby3 puncta at the same location as the Cy5 spots, and their departure), and dissociation of NPs (dissipation of Cy5 spots) were observed sequentially. For quantitative analysis, (G) a single z plane of the live cell imaging was tracked, and endosomal escape events induced by 50-nm and 400-nm NPs were compared. The arrows point to examples of endosomal escape events. The cumulative (total) endosomal escape events observed within the view (10–11 cells) during incubation are shown in (H) for 50-nm NPs and (I) for 400-nm NPs, while the present numbers of Gal8-mRuby3 puncta (i.e., ruptured endosomes) at the time of assessment are shown in (J) for 50-nm NPs and (K) for 400-nm NPs.

condition to that before the 2nd mixing step, whereas the pH is decreased to 3.0 with ionic strength reduced, thus the colloidal stability is re-introduced. Under transmission electron microscopy (TEM), each 400-nm NP appeared to be constructed by individual ~50-nm building block NPs (Figure 2A), supporting the mechanism described above. To further demonstrate the unique features of these stable vehicles, we carried out two experiments to compare the characteristics of the pDNA/PEIpro NPs at the optimal size of 400 nm *vs.* the 50-nm building block NPs.

Using our previously developed platform, cylindrical illumination confocal spectroscopy (CICS) technique,[26–28] we characterized pDNA payload capacity (i.e., pDNA copies per NP) in NPs with different sizes, using Cy5-tagged pDNA to prepare NPs that were

illuminated by a light sheet excitation in a tube flow. The fluorescent intensity of the emission from each NP was recorded for calculating the single-NP payload (Figure 2B and materials and methods). The fluorescent intensity distributions of pDNA/PEIpro NPs at the sizes of 50, 150, and 200 nm carrying 100% Cy5-pDNA are shown in Figure S1A, while those of NPs at the sizes of 300 and 400 nm carrying only 2% Cy5-pDNA to avoid signal saturation are shown in Figure S1B. Using a deconvolution algorithm that we developed previously[26] (materials and methods and Supplemental methods), we revealed that most of 50-nm building block NPs contained only one or two pDNA molecules per NP, whereas the 150-nm particles had a weight average payload of 11 pDNA per particle (Figure 2C). As the average particle size increased to 200, 300, and 400 nm, the weight average payload increased to 16, 92, and 221, respectively (Figure 2D). These values were significantly lower than densely packed solid particles with the same volume density of the 50-nm building block NPs. For example, the weight average of 221 pDNA/NP was less than one-half of the predicted payload for the 400-nm particles based on scaling with the volume (>500 copies per particle). This qualitatively agrees with the TEM images (Figure 2A), revealing the porous structure of the assembled 400-nm NPs. Given the findings[8] that most effective transfection occurs at an NP size of 400–500 nm and each cell received numerous NPs during transfection (Figures 2F and 2G), each cell would receive more than 1,000 copies of pDNA to achieve sufficiently high transfection efficiency.

The major advantage of 400-nm over 50-nm pDNA/PEIpro NPs was previously attributed to the more than 10-fold greater efficiency of cellular uptake, but not endosomal escape, based on the total intracellular fluorescent intensity of labeled pDNA measured using the high-content Cellomics analysis on fixed cells.[8] To further provide insights into the intracellular delivery mechanism that is dynamic in nature, we carried out live cell imaging using a high-resolution confocal microscope (Zeiss LSM800) to characterize cellular uptake and endosomal escape simultaneously. The B16F10 cell line transduced by galectin-8 (Gal-8)-mRuby3 gene[29] was used, in which the cytosolically distributed Gal-8 molecules bind to and thus concentrate around ruptured endosomes as a result of an escape event,[30] forming bright puncta inside the cell (Figure 2E). In tracking Cy5-tagged NPs, it was observed that abundant, if not most of 400-nm pDNA/PEIpro NPs effectively triggered Gal8-mRuby3 puncta shortly after uptake (Figure 2F, and the full recorded video is provided as Video S1), capturing the ongoing endosomal escape process.[31] As indicated by arrows in Figure 2F, Gal-8 puncta appeared near the NPs, and subsequently migrated away. After the escape, the originally dense Cy5 signal (presumably suggesting an intact pDNA/PEIpro NP structure) became dissipated slowly. It was not because NPs migrated to another Z plane since Figure 2F and Video S1 were obtained by stacking all Z planes from the confocal imaging (i.e., top-down view of a three-dimensional perspective). This is, therefore, believed to be the dissociation events of pDNA/PEIpro NPs to release pDNAs into the cytosol.

Compared with 50-nm NPs, 400-nm NPs showed a similar number of uptake events (Cy5 spots), but earlier and much greater successful rate of endosomal escape (mRuby3 puncta) as depicted by counted cumulative or at-the-moment endosomal escape events within the view including a similar number of cells at a single focal plane (Figure 2G, with statistics shown in Figures 2H–2k). At later time points nearing 4 h of incubation, most cells treated by 400-nm NPs contained 5–10 mRuby3 puncta per cell. This had made identification of new endosomal escape events impossible due to difficulty in distinguishing new puncta from existing puncta that moved to a new location from the last frame (the full recorded videos at this focal plane can be found in Videos S2 and S3, for 50-nm and 400-nm NPs, respectively). Based on this reporter assay and image analysis, the 400-nm NPs not only result in a higher cellular uptake level, but the NPs upon uptake have a higher efficiency in successful endosomal escape, compared with 50-nm NPs.

Scale-up production using the CIJ mixer and a dual syringe pump

Our first scale-up study was to convert the original single-injection mode of operation using a single syringe pump into a continuous flow set-up using two coupled syringe pumps equipped with check valves (Figure 3A). The check valve keeps a flow direction from the reagent reservoir toward the syringe when the pump retracts, and from the syringe toward the CIJ mixer when the pump pushes. Without increasing the flow rate significantly, we chose the original, previously described laboratory-scale CIJ mixer[32] (Figure 3B) (mixing chamber size = 27 μL) for this study. As illustrated in Figure 3C, the two pumps operate (i.e., push and retract) alternatively to keep a continuous flow. When mixing pDNA and PEIpro in CIJ mixer to produce stable, ∼50-nm building block NPs, the mixing quality has a major impact over the size and uniformity of the NPs.[19,20] We previously demonstrated that the mixing quality is directly dictated by flow rate, and there is a threshold flow rate above which mixing enters a fully turbulent regime, ensuring uniform distribution of molecules for complexation.[19] In this fully turbulent regime, the mixing is fast enough that the characteristic mixing time is shorter than the characteristic assembly time of pDNA/PEIpro NPs, and the flow rate therefore no longer affects the size and uniformity of the assembled NPs (Figure 3C, green region). To ensure consistent NP quality when the pumps switch directions, the pumps were programmed that (1) the retraction rate to aspirate pDNA and PEIpro solutions into the syringes was greater than that to push the solutions into the CIJ mixer; and (2) the pump with loaded syringes starts pushing at 0.25 s before the other pump stops pushing, creating an overlap during which both pumps are pushing. This program generated a surge of the total flow rate each time when the pumps switch directions, that prevents the mixing from leaving the fully turbulent regime, thus preventing fluctuations of NP quality during the entire run (Figure 3C). Compared with a single injection at 5-mL scale at 40 mL/min, pDNA/PEIpro NPs produced at 250-mL scale at 100 mL/min (the pumps switched directions for 6 times) only showed minimal increase in their z-average diameter (from 50.4 to 63.4 nm) and polydispersity index

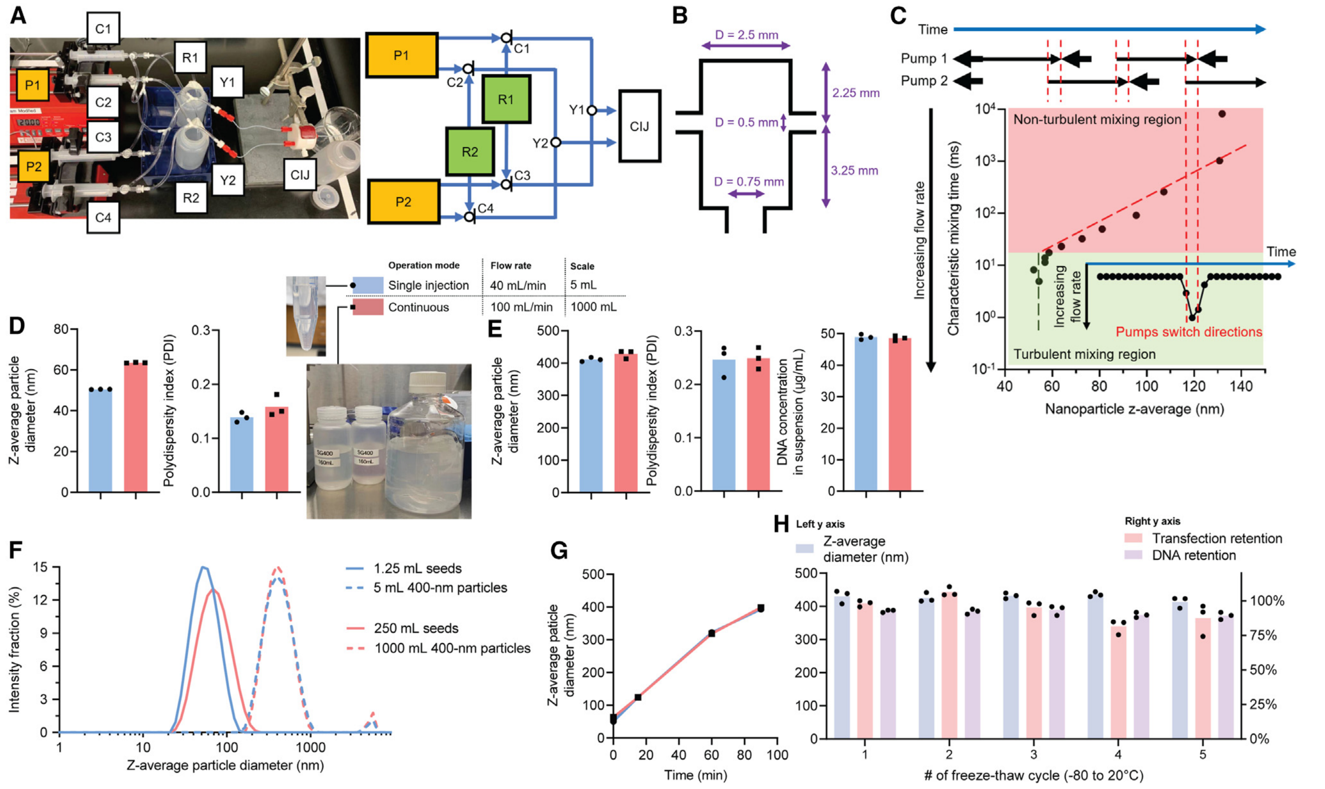

Figure 3. The continuous production set-up using a laboratory-scale CIJ mixer and dual syringe pumps

(A) Schematic of the production set-up. "P" denotes a syringe pump; "C" denotes a three-way check valve, which permits the flow direction of P → Y and R → P, but prohibits the flow direction of R → Y or Y → P/R; "R" denotes a reagent reservoir; and "Y" denotes a Y-shape connector and other necessary fluid fittings. (B) The dimension design of the CIJ mixer used in this set-up. (C) The operating mode of the dual syringe pumps that results in surges of the total flow rate to ensure fully turbulent mixing. Comparing production at the 5-mL scale using the conventional single-injection CIJ mixer with 1-L scale using the continuous production set-up, the z-average particle diameter and PDI assessed by DLS, and DNA concentration in the suspension assessed by Nanodrop are shown in (D) for the seed 50-nm pDNA/PEIpro NPs and (E) for 400-nm target NPs. The two production scales showed nearly identical (F) size distributions and (G) particle size growth curves. (H) The stability of 400-nm target NPs upon freeze-thaw cycles (from room temperature to −80°C). Note that (D) and (E) share the same legends with the grouping suggested by their different colors and types of symbol; and (F) and (G) share the same legends.

(PDI) (from 0.139 to 0.158) in the 1st mixing step to produce 400-nm NPs (Figure 3D). We proceeded using this scale-up setting to carry out mixing at a scale of 250 mL + 250 mL for the 2nd mixing step, and a scale of 500 mL + 500 mL for the 3rd mixing step. The final product had nearly an identical z-average diameter of around 400 nm, a PDI of 0.2–0.3, and a fully retained DNA concentration in the NP suspension (50 μg/mL), compared with the small batch production (Figures 3E and 3F). The minor difference in the average size of pDNA/PEIpro NPs after the 1st mixing step did not affect the quality of the 400-nm NP product. This is presumably due to the size growth mechanism under controlled solution conditions, such that the small NPs associated with each other in constructing 400-nm NPs. Specifically, with hundreds of building blocks needed to construct the sub-micron size (Figure 2D), the small initial size differences did not significantly alter the assembly kinetics (Figure 3G).

Effectively stabilized by excessive positive charges on NP surfaces and protected by a cryoprotectant (trehalose), the 400-nm pDNA/PEIpro NPs showed superior stability. We tested the size distribution and retention of both the DNA concentration in formulation and transfection capability (using luciferase pDNA blended into the pDNA cocktail at 2% w/w) of the product upon repeated freeze-thaw cycles (−80°C to ambient temperature). It was revealed that the NPs survived at least five cycles without significant changes in the physical properties and endured up to three cycles without a significant decrease in their transfection efficiency (Figure 3H). This feature can render these NPs as an off-the-shelf reagent for the cell transfection step.

Scale-up production using a dimension-enlarged CIJ mixer and peristaltic pumps

We then aimed to increase the production lot size of the 400-nm pDNA/PEIpro NPs by at least 10-fold. With difficulties in further increasing the flow rate using a syringe pump and the original CIJ mixer due to limitations in fluidic pressure, we designed a dimension-enlarged CIJ mixer with a mixing chamber volume of 0.73 mL

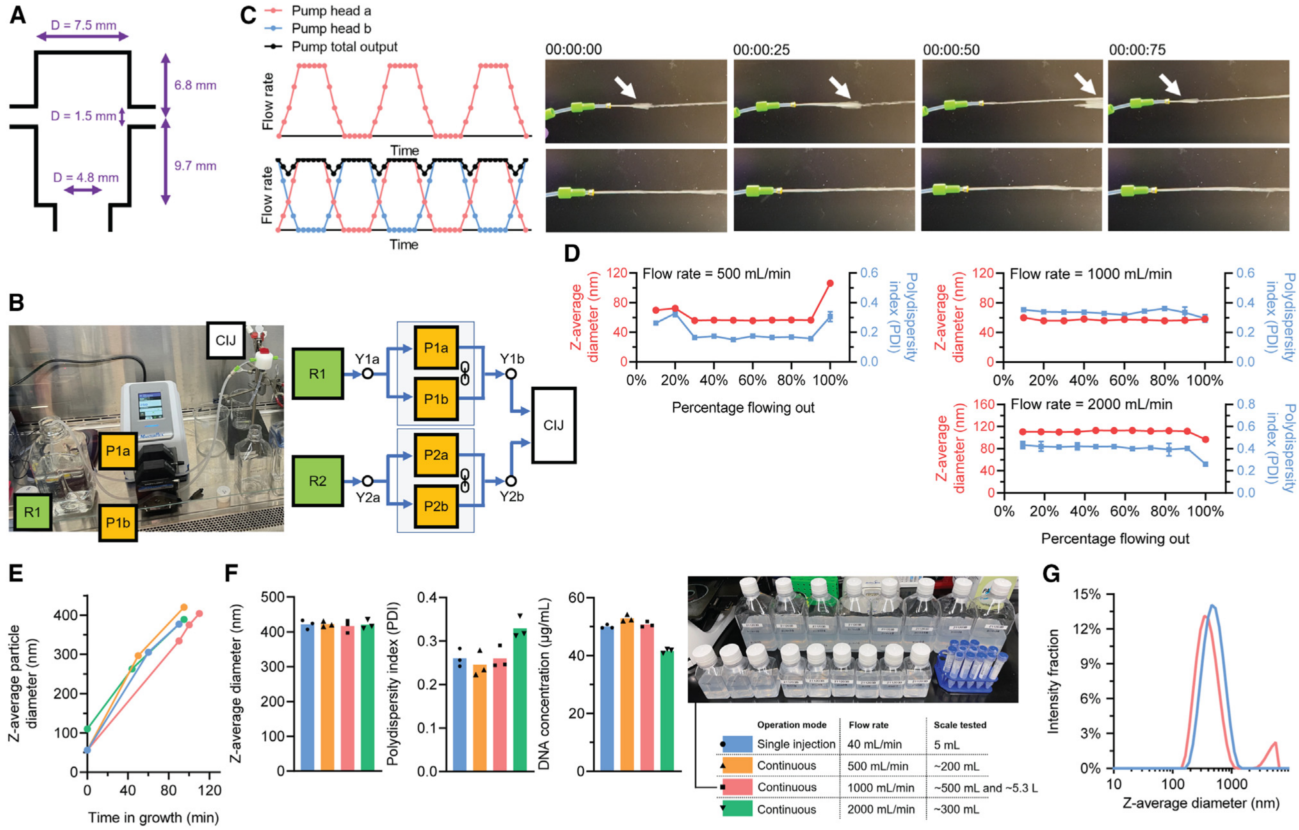

Figure 4. The continuous production set-up using a dimension-enlarged CIJ mixer and a pair of peristaltic pumps

(A) Dimension design of the CIJ mixer used at this set-up. (B) Process scheme of this continuous production set-up and half of an established example. "P" denotes a peristaltic pump; "R" denotes a reagent reservoir and "Y" denotes a Y-shape connector and other necessary fluid fittings. (C) Reduction of flow pulse by coupling two pump heads on a single peristaltic pump with a phase delay, which was easily observed in slow-motion recordings of flow outputs compared with a conventional single-head peristaltic pump. (D) Consistency of the quality of pDNA/PEIpro NPs (z-average particle size and PDI by DLS) produced during the entire production period with elevated total flow rates. (E) Comparison of the growth kinetics of seed pDNA/PEIpro NPs produced with different flow rates in all mixing steps. (F) Comparison of the quality of the final 400-nm NPs (z-average particle size and PDI by DLS, and DNA concentration in suspension by NanoDrop) produced by different flow rates in all mixing steps. (G) Comparison of the size distribution of the final 400-nm NPs (by DLS) produced at 5-mL and 5.3-L scales. Note that (E) and (F) share the same legends, with the grouping suggested by different colors and types of symbols.

(Figure 4A) and switched to dual-peristaltic pumps (Figure 4B, showing only one side of the inputs into the CIJ). This entire set-up, with 2-L reagent bottles used as reservoir and collection containers and all supporting holders and supplies in our experiments, fits into a standard 6-ft biosafety cabinet (BSC), which allowed us to produce a target scale of 5 L under a sterile condition. Because the target 400-nm pDNA/PEIpro NPs could not be terminally sterilized using a 0.22-μm filter, all reagents were purchased or prepared in a sterile form; and all supplies were autoclaved before entering the BSC. The operation surface was UV-treated for 30 min within the BSC, and all production procedures followed a standard Biosafety Level 2 containment protocol within the BSC to avoid contamination. One challenge in using a conventional peristaltic pump lies with the flow pulse naturally embedded in the mode of fluid displacement during pump rotations, when drastic flow rate change occurs (Figure 4C, top) (observed under a flow rate of 500 mL/min). This introduces inconsistency to the flow rates of the jets introducing pDNA and PEIpro solutions and increases the risk of dropping the flow rate below the critical threshold needed to maintain the turbulent mixing condition. To address this challenge, we stacked two pump heads (Masterflex L/S Easy-Load II) onto a single peristaltic pump drive (Figure 4B). There was a phase delay of 45° between these two four-roller pump heads that effectively smoothed the flow rate (Figure 4C, bottom) (observed with a pump drive rate of 250 mL/min that is 500 mL/min when two pump heads combined). Even though complete removal of flow pulse was difficult, as occasional fluctuations were captured by a slow-motion camera (Figure 4C, bottom), the continuous flow was maintained steadily at a targeted flow rate of 500–2000 mL/min, which significantly increased the Reynolds number (*Re*) operated in this larger CIJ mixer (Table 1) that ensured the mixing regime is maintained within the fully turbulent region.

For the 1st mixing step to produce building block pDNA/PEIpro NPs, a reference batch with the original laboratory scale (5-mL production

Table 1. Evaluation of turbulent mixing by different devices and flow rates

Flow rate (mL/min)	CIJ mixer (chamber volume, μL)	Outlet diameter (mm)	Outlet speed (mm/s)	*Re*[a]
40	27	2.5	135.8	339.5
100	27	2.5	339.5	848.8
500	730	7.5	188.6	1,414.7
1,000	730	7.5	377.3	2,829.4
2,000	730	7.5	754.5	5,658.8

[a]Defined as the product of fluid density, flow speed, and outlet diameter, divided by the dynamic viscosity of the fluid. All calculations assumed pure water (25°C) as the fluid.

at 40 mL/min) typically yields a z-average size of approximately 50 nm and a PDI of approximately 0.14. To test the steadiness of the flow mixing, mixing of pDNA and PEIpro was carried out with large volumes, and the NPs were collected into aliquots sequentially. A flow rate of 500 mL/min using the scale-up set-up generated a nearly identical size, while PDI only increased marginally to 0.164 on average during the steady phase of a batch of 200 mL, excluding the initial collection aliquots when a steady mixing had not been established yet, and the final aliquot during the pump shut-down (Figure 4D, top left). A further increase of flow rate to 1 L/min in a 500-mL batch production did not alter the NP size; however, the PDI increased slightly to 0.337 on average (Figure 4D, top right).

Table 2. QC matrix for 5-L batch produced at a flow rate of 1 L/min using dimension-enlarged CIJ and peristaltic pumps

	This scale-up batch	A reference laboratory-scale batch
Target size	400 nm	
Target DNA concentration	50 μg/mL	
Production mode	peristaltic pumps with dimension-enlarged CIJ mixer	single syringe pump with laboratory-scale CIJ mixer
Production flow rate	1 L/min	20 mL/min
Production volume	∼5.3 L	5 mL
Freshly prepared samples		
Z-average diameter (DLS)	408.6 ± 15.9 nm	397.2 ± 3.2 nm
PDI (DLS)	0.339 ± 0.079	0.238 ± 0.023
DNA in suspension (NanoDrop)	46.0 ± 0.8 μg/mL	49.4 ± 1.4 μg/mL
Upon freeze-thaw cycle		
Z-average diameter (DLS)	421.7 ± 15.2 nm	409.6 ± 0.6 nm
PDI (DLS)	0.354 ± 0.041	0.231 ± 0.039
DNA in suspension (NanoDrop)	45.6 ± 0.9 μg/mL	50.0 ± 1.8 μg/mL

Increasing the flow rate to 2 L/min in a 300-mL batch scale significantly increased the average NP size by nearly 2-fold to 111.6 nm on average, and the PDI reached 0.411 (Figure 4D, bottom right). In evaluating the size growth upon the 2nd mixing step (Figure 4E), and the 3rd mixing step to produce stabilized 400-nm pDNA/PEIpro NPs (Figure 4F), the same flow rates were used as that of the 1st mixing step, using building block NPs prepared after the 1st mixing step. All NPs generated with different flow rates showed a similar size growth kinetics; however, the lower uniformity of the building block NPs generated with 2 L/min led to a lower quality of the final product. Even though the target size of 400-nm was achieved, the final product had a higher PDI of 0.329 and a loss of 16.8% DNA during the process (Figure 4F). Therefore, we chose a flow rate of 1 L/min to produce a validation batch with a target lot size of 5 L of 400-nm pDNA/PEIpro NPs.

This lot was successfully produced with a total volume of ∼5.3 L containing ∼0.265 g total pDNA. No contamination was detected in an antibiotics-free suspension cell culture upon addition of NPs at 1 μg pDNA/mL, suggesting that good sterile conditions were maintained in this production. The entire process was planned into 3 days: On the 1st day, all necessary instruments and supplies were autoclaved, and set up in the BSC, and all solutions were prepared. On the 2nd day, the three mixing steps were carried out within ∼4 h. The final recorded size growth time for this lot was 105 min. The produced NPs were aliquoted and frozen directly into −80°C with a volume of either 500 mL in 1-L bottles, 125 mL in 250-mL bottles, or smaller aliquots (Figure 4F, photo insert). On the 3rd day, QC was performed with thawed aliquots (Table 2, with the NP size distribution reported in Figure 4G). The entire process is modular and can be easily modified based on the shift schedule. This lot of 400-nm pDNA/PEIpro NPs met the scale criteria for LVV production tests as it is comparable with the scale required for a typical LVV production in the field. More important, there is no lot size limit in further scaling up the process beyond 5 L using the 1 L/min flow rate.

Validation of scale-up stable pDNA/PEIpro NPs in production of LVVs

Both the 1-L and 5-L lots were shipped on dry ice to 2seventy bio, Inc., and stored at −80°C. With the 1-L lot, the on-bench stability upon thawing at ambient temperature was tested using Ambr 15 cell culture system at a cell culture volume of 15 mL to produce LVVs. A major limitation of the conventional method of transfection in LVV production (Figure 1A) is the inconsistent yield associated with the timing of dosage within a short period of time of incubation of the pDNA and PEIpro mixture (Figures 5A and S2A). In contrast, the thawed 400-nm NPs under ambient temperature maintained their ability to effectively transfect the production cell line for at least 3 h, producing LVVs at a consistent, standard titer characterized by HOS cell line assay (Figures 5B and S2B). In a separate study on Ambr 15, the 1-L and 5-L batches were compared against the control, which used the conventional transfection protocol in which pDNA and PEIpro are mixed immediately before dosage, at different viable cell densities (VCDs) at seeding. The pDNA dose of NPs was controlled to be

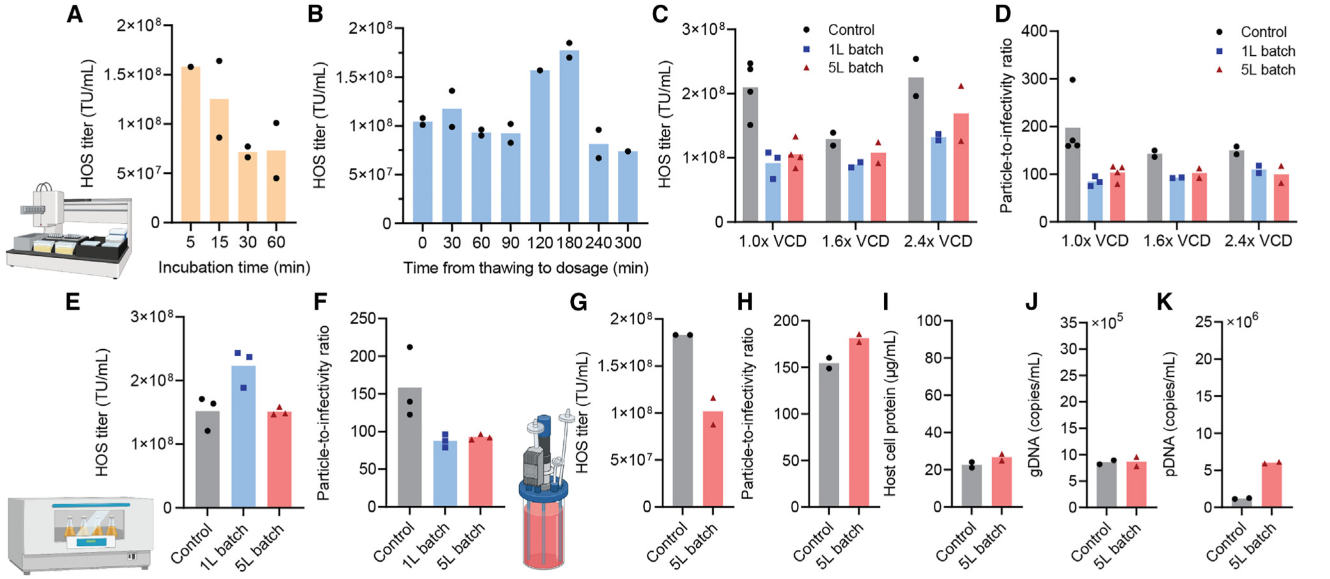

Figure 5. Performances of scale-up batches of pDNA/PEIpro NPs in production of LVVs

LVV production tests using (A—D) AMBR15, (E and F) shake flask culture with a culture volume of 100 mL, or (G—K) 2-L stir-tank bioreactors. (A) The harvest LVV titer significantly decreased within 60 min upon mixing of pDNA and PEIpro in the conventional method, but (B) did not significantly decrease, even after 180 min standing at ambient temperature upon thawing the 1-L lot samples of stable 400-nm pDNA/PEIpro NPs. The LVV production performances were characterized by (C, E, and G) the overall harvest titer and (D, F, and H) the P:I ratio. For the 2-L bioreactor production test, impurities were also characterized in terms of (I) residual host cell protein, (J) residual genetic DNA (gDNA), and (K) residual pDNA. In (C) and (D), VCD, for which 1× represents a standard condition, and NP dose was proportional to the VCD level.

proportional to the seeding VCDs. The two lots of NPs yielded similar titers per unit volume across different VCD levels (Figure 5C). The thawed NPs from both lots yielded on average 43.7%–83.9% LVV titers compared with the control (Figures 5C and S2C), which was consistent with our previously published results on stable NPs at a 100-mL scale.[8] Another feature of these NPs, consistent with the smaller batch results,[8] was a lower particle-to-infectivity (P:I) ratio (defined as a ratio of the capsid protein p24 quantification[33] over the infectious titers by the HOS cell-based assay as described in the materials and methods) (Figure 5D). This implies that a greater proportion of the viral RNAs and proteins produced by transfection assembled into functional LVVs. This might be a result of improved co-expression of multiple species of pDNAs by stable 400-nm pDNA/PEIpro NPs.[34] These results confirmed that the NP lots produced at larger scales carried the same properties in this testing platform.

We also tested the activity of these two lots of NPs using a shake flask culture with a culture volume of 100 mL. In this culture setting, both 1-L and 5-L lots of stable NPs yield comparable infectious titers as that from the control which used the conventional transfection protocol (Figure 5E), and lower P:I ratios were also confirmed (Figure 5F). The 1-L lot was tested after 10 months of storage in −80°C in this experiment, indirectly suggesting shelf stability of at least 10 months for these NPs.

We then tested these NPs using 2-L stir-tank bioreactors at 2seventy bio, Inc. The on-bench stability as well as high pDNA concentration (50 μg/mL) of these NPs in suspension enable more easily implemented control over dosage. Using the 1-L lot NPs, the NP doses of 1×, 1.6×, or 2× of the standard dose (1 μg total pDNA/mL) were tested at a seeding VCD of 1×, 1.4× or 1.6×. We did not find qualitative differences in the production yield or P:I ratio among these conditions tested, while the 1.6× dose combined with 1.6× VCD seemed to produce the highest harvest titer (Figures S2E, S2F, and S2G). Under the standard production conditions of 1× dose and 1× VCD, the 5-L lot NPs yielded an infectious LVV titer that was about 50% of the control (Figures 5G and S2H) and the same P:I ratio (Figure 5H). The differences between the 15-mL Ambr and 2-L bioreactor transfection settings might be due to the differences in how NPs are mixed with the cell culture through liquid handling across scales, as well as differences in the modality used for medium exchange (materials and methods), which might have altered the cellular uptake efficiency. In assessment of impurities, the 5-L lot NPs did not generate a different level of host cell protein (Figure 5I), or genetic DNA (gDNA, Figure 5J) compared with the control. The gDNA concentration represents the amount of HEK293T genome DNA present in the cell culture after centrifugation and is a standard quality attribute as part of process control at each step to ensure that the final impurity level meets the process criteria. While an increase in residual pDNA (from the transfection particles) (Figure 5K) to 6.0 × 10^6 copies/mL from the control level of 1.2 × 10^6 copies/mL was observed in the 5-L lot NP group, this impurity level was within the typical range that other process changes such as transfection scale, timing, and

modality could generate. During the LVV manufacturing process, the downstream purification steps to generate the final LVV product typically reduce such impurity by one to two magnitudes, and the elevated residual pDNA level in this study is still within the typical purification capability. However, further optimization in the cell culture and transfection processes are warranted to further decrease the amount of harvest impurities. These results in general validated the performances of scale-up produced 400-nm pDNA/PEIpro NPs in an industrial setting of LVV productions.

DISCUSSION

Kinetics considerations for implementing scale-up modalities in multi-step mixing to produce 400-nm pDNA/PEIpro NPs

Analyzing kinetics in the multiple mixing steps was one of the most important considerations when establishing the scale-up production set-ups for the method to produce 400-nm pDNA/PEIpro NPs (Figure 1D).

The 1st mixing step, i.e., mixing of pDNA and PEIpro solutions, concerns complexation between pDNA and PEIpro molecules to form NPs as building blocks for the following steps. In our previous investigations, we estimated that the characteristic assembly time for pDNA and PEI to form stable NPs is on the scale of tens of milliseconds.[19] Using the CIJ mixer shown in Figure 3B, a total flow rate higher than 40 mL/min that renders a *Re* of greater than 339.5 (Table 1), ensures fully turbulent mixing of pDNA and PEIpro solutions with a characteristic mixing time shorter than the characteristic assembly time.[19] A global effect is that the distribution of pDNA and PEIpro through diffusion is uniform before NP assembly largely starts, thus resulting in homogeneity in the assembled NP size. Within this fully turbulent mixing regime, an increase in or local fluctuations of the flow rate would not significantly impact mixing quality, thus generating pDNA/PEIpro NPs with a consistent quality. In this study, when the original CIJ mixer (Figure 3B) was used in combination with dual syringe pumps, we used a greater total flow rate of 100 mL/min (*Re* = 848.8) to ensure the NPs were generated within the turbulent mixing regime and programmed the two pumps to have a brief overlap period of pushing when one pump was about to switch direction to ensure the flow rate never dropped below the threshold; when a dimension-enlarged CIJ mixer (Figure 4A) was used in combination with dual peristaltic pumps, the significantly increased total flow rate to 0.5, 1, and 2 L/min overrides the dimension enlargement and gave increased *Re*s that were much higher than those within the original CIJ device (Table 1). Notably, the 50-nm pDNA/PEIpro NPs generated at 0.5 L/min flow rate (*Re* = 1414.7) had almost identical characteristics as NPs generated at laboratory scale at 40 mL/min (Figure 4D), supporting the kinetics theory discussed above. When the total flow rate increased to 1 L/min, the uniformity of the pDNA/PEIpro NPs dropped while 2 L/min generated NPs with a larger size and even worse uniformity. We attribute these sub-optimal performances to inherent fluidic instability associated with extremely high *Re*s and/or with the peristaltic pumps, while more investigations are needed to reach a conclusion.

The 2nd and 3rd mixing steps mix pDNA/PEIpro NPs with buffers to initialize and quench NP size growth, respectively (Figure 1D). The high liquid volume in these mixing steps in this study (up to 5.3 L), and in further scale-up practices in the future, requires a significant amount of time to flow, even with a high total flow rate. Reducing heterogeneity of NPs generated by temporal differences in flowing into the 2nd and 3rd mixing steps is of great interest. In our previous proof-of-concept study detailing the pDNA/PEIpro NP growth mechanisms, we found that the ionic strength of the assembly buffer (i.e., the buffer to be mixed with stable 50-nm building blocks, Figure 1C) positively correlates with the NP size growth kinetics.[8] Therefore, the ionic strength of the assembly buffer in this study was tuned at a low level to give a slow NP size growth kinetics, that it needed roughly 2 h to grow from 50 to 400 nm. This rendered the time to flow 50-nm NPs and the assembly buffer into the 2nd mixing step, and the time to flow growing NPs and the stabilization buffer into the 3rd mixing step negligible in generating any appreciable differences in the final 400-nm NP product. It gave sufficient time between the 2nd and 3rd mixing steps to collect NPs, prepare the fluidics, and carry out QC (in this study, dynamic light scattering [DLS] size measurements to monitor the NP size growth) to achieve better engineering control over the process. In addition, it renders the entire process operator-independent and lot-to-lot reproducible to generate pDNA/PEIpro NPs with consistent quality. Indeed, the implementation of this slow NP growth kinetics into the 5.3-L production yielded 400-nm pDNA/PEIpro NPs with almost identical characteristics as compared with those produced at a laboratory scale at 40 mL/min (Figures 4F and 4G).

Forward looking with shelf-stable and on-bench stable pDNA/PEIpro NPs

To a certain extent, the space for further optimizing the industrial practices for transient transfection, and further increasing viral vector production yield through more efficient transient transfection has been partially limited by the unstable nature of the pDNA/PEIpro NPs upon reagent mixing (Figure 1A). This is because any liquid handling procedures designed for scale-up production must put a high priority on fitting within a tight time window, before considering other parameters that can potentially impact transfection efficiency. Our developments of shelf-stable and on-bench stable pDNA/PEIpro NPs break such strategic restraint. The stability data shown in this study (Figure 5B) as well as in our previous study[8] indicate that the 400-nm pDNA/PEIpro NPs are stable upon thawing from storage for at least 6 h at ambient temperature. This feature may give rise to new designs and strategies to use these NPs in bioreactor cell cultures. For example, some originally impossible optimizations may now become possible such as to investigate how to optimize the contact between NPs and cells within the bioreactor through fluidic engineering, and to optimize the concentration of the stable NPs. This method would also be compatible with conventional optimization parameters such as fine-tuning the pDNA ratio and the PEIpro-to-pDNA ratio,[35] as well as incorporation of transfection enhancers and design of experiment approaches.[36]

In summary, we established a stepwise mixing protocol to assemble pDNA/PEIpro NPs at the desired size of 400 nm for transfecting production cell lines to produce LVVs, successfully generated two scale-up production lots at 1-L and 5-L scales and assessed the quality of these NPs in LVV production tests. These results demonstrated the translation potential of this NP assembly technique to produce pDNA/PEIpro NPs that can be quality tested and stored. The unique features of these NPs include superior shelf stability and on-bench stability after thawing. The shelf stability enabled that the production of these NPs can be separated from the transfection procedures, and QC can be applied to the transfection particles to provide additional robustness of the entire LVV production process. The on-bench stability at ambient temperature at minimum provides additional operational benefits, including reduced sensitivity to timing and more flexible workflow for cell transfection. We expect it to bring more potential benefits in larger scale viral vector production developments, particularly in bioreactor dosing set-ups. The platform may also find broad applications in the production of other biological agents, such as adeno-associated virus and protein therapeutics.

MATERIALS AND METHODS

Materials and reagents

The pDNAs used during the testing of scale-up designs were purchased from Aldevron (gWiz-luciferase pDNA, Cat. #5001); pDNAs used in the validation experiments at different scales for LVV production were provided by 2seventy bio, Inc. and used as received. The PEIpro was purchased from Polyplus Transfection SA. Nuclease-free water (Invitrogen, Cat. #AM9932) was used to dilute the pDNA and PEIpro and to prepare all the solutions used. The DNA concentration prior to any mixing step was 400 μg/mL. Each of the 1st, 2nd, and 3rd mixing steps decreases the overall DNA concentration in suspension by 2-fold, to 200, 100, and 50 μg/mL, respectively. For the 2nd mixing, an assembly buffer to induce NP size growth was mixed with stable building block NPs at 1:1 volume ratio. The assembly buffer was described in our previous report[8] (pH 7.0, containing chloride and phosphate salts). For the 3rd step mixing, a stabilization buffer to quench NP growth was mixed with the growing NPs at a 1:1 volume ratio. It contains an acidic pH buffer to re-protonate amine groups on PEIpro molecules, thus re-establishing charge repulsion as a source of stability,[8] as well as the cryoprotectant of trehalose (at a concentration of 19% w/w).

Scale-up production set-up using a laboratory-scale CIJ mixer and dual syringe pumps

The two syringe pumps used in this set-up were purchased from New Era Pump Systems (Cat. #NE-4000) with recommended firmware upgrade, connected by a communication cable (Cat. #CBL-DUAL-3) following the manufacturer's protocol. The check valves were purchased from the same manufacturer (Cat. #P-DKIT). The CIJ mixer used in this set-up has been fully described in our previous publications.[19,32]

Scale-up production set-up using dimension-enlarged CIJ mixer and peristaltic pumps

The peristaltic pump drives and heads were purchased from Avantor (Masterflex L/S MasterSense, Cat. #07526-10, and Masterflex L/S Easy-Load II, Cat. #77200-30). Two pump heads were stacked on a single pump drive following the manufacturer's protocols. The dimension-enlarged CIJ device was manufactured by computer numerical control machining using Delrin acetal resin (DuPont, Cat# FG150 NC010). Fluidic connections were established using PuriFlex tubing (Masterflex Cat. #96419-16), PFA hard tubing (IDEX Fluidics, Cat. #1641), Y connectors (Masterflex Cat. #40726-43), and adaptors (Masterflex Cat. #21941-48 and IDEX Fluidics Cat. #P-359).

QC steps during and after the manufacturing of 400-nm pDNA/PEIpro NPs

While the kinetics of NP growth was consistent across batches and scales, the real-time NP size during the size growth period before the 3rd mixing was monitored by sampling 100 μL of the NP suspension on a DLS instrument (Malvern, Model ZS-90). The size was measured at a 90° detection angle in 10 runs with a measurement time of 10 s for each run. This sampling was carried out repeatedly (every 5 min) starting from 75 min after the 2nd mixing step. The 3rd mixing step was initiated within 5 min when the z-average diameter given by DLS reached 400 nm. Approximately 30 min after the 3rd mixing to stabilize the NPs, The same DLS measurement was carried out again, and the NPs were sampled three times and measured independently. Besides the z-average diameter, the PDI and the intensity-based size distribution were also recorded. To assess total pDNA concentration in the NP suspension, 2 μL of the sample was measured on a NanoDrop instrument characterizing absorbance at 260 nm (A_{260}). Our previous work (*c.f.* Figure S4 in ref.[19]) identified that, once pDNA is complexed with PEI, its concentration-to-A_{260} coefficient increases to 57.25 from that of naked pDNA of 50. This coefficient was used to derive the pDNA concentration in the NP suspension.

Characterization of pDNA payloads in pDNA/PEIpro NPs

The CICS method features a flow set-up and single-photon sensitivity and high mass detection efficiency, which quantitatively resolves the fluorescent intensity emitted from individually labeled NPs. Detailed instrument set-up and experimental procedures were described in our previous report, in which we characterized mRNA lipid NPs.[26] For pDNA/PEIpro NPs, the pDNAs were covalently labeled by Cy5 following a previous report[37] and used with a 100% weight ratio for 50-, 150-, and 200-nm NPs, or 2% for 300- and 400-nm NPs. The reduced weight ratio of Cy5-pDNA for 300- and 400-nm NPs was required to avoid signal saturation. The Cy5-pDNA/PEIpro NPs at different sizes were diluted by 1 mM HCl with 0.5% polyvinylpyrrolidone (PVP) (360 kDa, Sigma-Aldrich, Cat. #P5288). PVP was used as a dynamic coating additive to minimize the surface adsorption in the microfluidic channel. The fluorescence detection was performed in a fused silica capillary (Polymicro Technologies) with a 10-μm nominal inner diameter and 55-cm effective length. The NPs were injected continuously into the capillary at 16 Psi (~1 mm/s) and

screened for 20–30 min. The same procedure was followed for the collection of pDNA-Cy5 single-molecule fluorescence. Thresholding and baseline were applied to the raw fluorescence signals to identify the single-NP events. For each event, the fluorescence burst size, height, width, and retention time were recorded. The fluorescence burst size (number of photons) was used to plot the fluorescent intensity distribution of each NP species (Figure S1). A deconvolution algorithm[27] was used to calculate the pDNA payload in pDNA/PEIpro NPs based on the fluorescence distributions obtained. The analysis details for this NP series are described in the Supplemental methods.

Live cell imaging for characterization of the cellular uptake and endosomal escape processes of pDNA/PEIpro NPs at different sizes

The Gal-8-based reporter assay to characterize endosomal escape was originally described by Kilchrist et al.,[30] and we previously used the B16F10 cancer cell line with the Gal-8-mRuby3 gene integrated using the PiggyBac transposon/transposase system[29] to perform endosomal escape assay on fixed cells.[8] For live cell imaging, the cells were seeded onto a four-well Nunc Lab-Tek II #1.5 chamber slide (Thermo Fisher Scientific, Cat. #155382) at a density of 0.1 million cells/mL, at 24 h before the experiments. The 50- or 400-nm pDNA/PEIpro NPs carrying 10% Cy5-labeled pDNA were diluted by Opti-MEM reduced serum medium (Gibco, Cat. #31985062) to a concentration of 1 μg pDNA/mL. The original full culture medium, high-glucose DMEM supplemented by 10% fetal bovine serum, was then completely replaced by this NP-containing medium. Then the chamber slide was mounted on a Zeiss LSM800 confocal microscopy equipped with a cell culture chamber. The imaging was carried out under a 63× lens from approximately 30 min after mounting for a total of 4 h. A 101.41 μm × 101.41 μm area was imaged; and a z stack was performed with a slide thickness of 0.27 μm.

LVV production tests

Stable NPs produced at different scales were stored at −80°C and shipped on dry ice to 2seventy bio, Inc., where the NPs were stored under −80°C until use. In-house suspension-adapted HEK293T cells were seeded into 15-mL Ambr 15 micro-bioreactors (Sartorius Stedim Biotech) at one of three VCDs: a standard VCD (1× VCD), or an elevated level (1.6× or 2.4× VCD) of the control VCD. After a predetermined amount of time, medium exchange was performed, and the culture was then transfected with NPs at an equivalent DNA concentration of 1 μg/mL (for standard VCD reactors) by addition of corresponding volumes of the thawed stable NPs at 50 μg pDNA/mL using the automated pipetting of the liquid handler. We targeted the 1.6× and 2.4× VCD reactors for a 1.6 and 2.4 μg/mL equivalent DNA concentration, respectively. Parallel transfections using the conventional method (Figure 1A) were performed using the same process conditions. After a predetermined incubation time with the NPs, medium exchange was performed again. After another predetermined incubation time, the microbioreactor cultures were harvested and clarified by centrifugation at 500×*g* for 5 min. The supernatants were sampled for analysis. Infectious titer (harvest titer) results in titer units per milliliter were determined by transduction of HOS cells following protocols developed by 2seventy bio, Inc. Briefly, after HOS cells reached desired density, they were transduced with multiple dilutions of harvested LVVs. After a predetermined culture time, HOS genomic DNA was extracted, and PCR quantifying the transfer gene was performed to calculate the transducing units. The capsid protein p24 (ng/mL) was assessed by ELISA (Alliance HIV-I p24 ELISA Plate Kit, PerkinElmer). The p24 value was an indicator of total LVV particles, and the ratio between infectious titer and p24 detected was derived to the P:I ratio.

The same suspension adapted HEK293T cell line was used and seeded into a 2-L stir-tank bioreactors. When cells reached a predetermined density, medium exchange was performed, and the culture was then transfected with NPs at an equivalent DNA concentration of 1 μg/mL by addition of corresponding volumes of the thawed NPs at 50 μg pDNA/mL using a peristaltic pump. After a predetermined incubation time with the NPs, medium exchange was performed again. After another predetermined incubation time, the cell cultures in the 2-L bioreactors were harvested and clarified by centrifugation at 500×*g* for 5 min, and the supernatants were sampled for analysis. Infectious titer (harvest titer) and p24 results were then determined using the methods described above. After harvest, the viral genetic materials were purified by the QIAamp 96 Virus QIAcube HT kit (Qiagen) following the manufacturer's protocol, and the residual HEK293T genome DNA (as in Figure 5J) and pDNA (as in Figure 5K) were quantified by a PCR protocol developed by 2seventy bio, Inc. The host cell protein (μg/mL) concentration was measured by ELISA (HEK 293 HCP ELISA Kit, Cygnus Technologies).

DATA AND CODE AVAILABILITY

All data to support the conclusions of this manuscript are available in the main text or the Supplemental Information associated with this manuscript.

SUPPLEMENTAL INFORMATION

Supplemental information can be found online at https://doi.org/10.1016/j.omtm.2024.101194.

ACKNOWLEDGMENTS

This work was supported by 2seventy bio, Inc. and National Cancer Institute (NCI) through a grant (R01CA281143) to H.-Q.M. The authors would like to thank Nolan D. Sutherland for input on the overall study design; David R. Wilson and Prof. Jordan J. Green from the Department of Biomedical Engineering at Johns Hopkins University for establishing the B16F10-Gal8-mRuby3 cell line; Stipe Iveljic and Rich Middlestadt from the Johns Hopkins University, Whiting School of Engineering Machine Shop for manufacturing of the CIJ devices; Yicheng Zhang from the Department of Materials Science and Engineering at Johns Hopkins University for assistance in formulation experiments; Sarvani Manne, Chiu-Ping Liu, Lucille Altman-Sagan, and David Kotlikoff from the vector process development team at 2seventy bio, Inc. for supporting LVV production experiments; and the analytical team at 2seventy bio, Inc. for characterization of the yield and quality of the LVVs produced in this study.

AUTHOR CONTRIBUTIONS

Y.H. designed and developed the scale-up production methods, as well as formulated and characterized the pDNA/PEIpro NPs; S.L. and T.-H.W. carried out the single-NP pDNA payload characterizations; J.L. and Y.Z. assisted scale-up NP batch productions and quality testing; B.A.E. and the 2seventy bio Inc. vector development team carried out transfection experiments in producing LVVs and coordinated assessments of the vector yield and purity; Y.H., B.A.E., T.G., and H.-Q.M. carried out the overall study design; T.G. and H.-Q.M. supervised the study. Y.H. drafted the manuscript; and B.A.E., T.G., and H.-Q.M. revised the manuscript with input from all the other authors.

DECLARATION OF INTERESTS

The authors declare the following competing financial interests: Y.H., Y.Z., and H.-Q.M. are inventors of patent applications on the NP assembly method described in this paper, filed through, and managed by Johns Hopkins Technology Ventures. B.A.E. and T.G. are employees of 2seventy bio, Inc., and hold company stock.

REFERENCES

1. Martínez-Molina, E., Chocarro-Wrona, C., Martínez-Moreno, D., Marchal, J.A., and Boulaiz, H. (2020). Large-scale production of lentiviral vectors: Current perspectives and challenges. Pharmaceutics *12*, 1051.
2. Wang, D., Tai, P.W.L., and Gao, G. (2019). Adeno-associated virus vector as a platform for gene therapy delivery. Nat. Rev. Drug Discov. *18*, 358–378.
3. Merten, O.-W., Hebben, M., and Bovolenta, C. (2016). Production of lentiviral vectors. Mol. Ther. Methods Clin. Dev. *3*, 16017.
4. Grieger, J.C., Soltys, S.M., and Samulski, R.J. (2016). Production of recombinant adeno-associated virus vectors using suspension HEK293 cells and continuous harvest of vector from the culture media for GMP FIX and FLT1 clinical vector. Mol. Ther. *24*, 287–297.
5. Durocher, Y., Pham, P.L., St-Laurent, G., Jacob, D., Cass, B., Chahal, P., Lau, C.J., Nalbantoglu, J., and Kamen, A. (2007). Scalable serum-free production of recombinant adeno-associated virus type 2 by transfection of 293 suspension cells. J. Virol. Methods *144*, 32–40.
6. Shupe, J., Zhang, A., Odenwelder, D.C., and Dobrowsky, T. (2022). Gene therapy: Challenges in cell culture scale-up. Curr. Opin. Biotechnol. *75*, 102721.
7. van der Loo, J.C.M., and Wright, J.F. (2016). Progress and challenges in viral vector manufacturing. Hum. Mol. Genet. *25*, R42–R52.
8. Hu, Y., Zhu, Y., Sutherland, N.D., Wilson, D.R., Pang, M., Liu, E., Staub, J.R., Berlinicke, C.A., Zack, D.J., Green, J.J., et al. (2021). Size-controlled and shelf-stable DNA particles for production of lentiviral vectors. Nano Lett. *21*, 5697–5705.
9. Pezzoli, D., Giupponi, E., Mantovani, D., and Candiani, G. (2017). Size matters for in vitro gene delivery: Investigating the relationships among complexation protocol, transfection medium, size and sedimentation. Sci. Rep. *7*, 44134.
10. Ogris, M., Steinlein, P., Kursa, M., Mechtler, K., Kircheis, R., and Wagner, E. (1998). The size of DNA/transferrin-PEI complexes is an important factor for gene expression in cultured cells. Gene Ther. *5*, 1425–1433.
11. Kasper, J.C., Schaffert, D., Ogris, M., Wagner, E., and Friess, W. (2011). The establishment of an up-scaled micro-mixer method allows the standardized and reproducible preparation of well-defined plasmid/LPEI polyplexes. Eur. J. Pharm. Biopharm. *77*, 182–185.
12. Chaudhary, N., Weissman, D., and Whitehead, K.A. (2021). mRNA vaccines for infectious diseases: Principles, delivery and clinical translation. Nat. Rev. Drug Discov. *20*, 817–838.
13. Sealy, A. (2021). Manufacturing Moonshot: How Pfizer Makes its Millions of Covid-19 Vaccine Doses. CNN News (April 2, 2021). https://edition.cnn.com/2021/03/31/health/pfizer-vaccine-manufacturing/index.html.
14. Shepherd, S.J., Warzecha, C.C., Yadavali, S., El-Mayta, R., Alameh, M.-G., Wang, L., Weissman, D., Wilson, J.M., Issadore, D., and Mitchell, M.J. (2021). Scalable mRNA and siRNA lipid nanoparticle production using a parallelized microfluidic device. Nano Lett. *21*, 5671–5680.
15. Belliveau, N.M., Huft, J., Lin, P.J., Chen, S., Leung, A.K., Leaver, T.J., Wild, A.W., Lee, J.B., Taylor, R.J., Tam, Y.K., et al. (2012). Microfluidic synthesis of highly potent limit-size lipid nanoparticles for in vivo delivery of siRNA. Mol. Ther. Nucleic Acids *1*, e37.
16. Johnson, B.K., and Prud'homme, R.K. (2003). Chemical processing and micromixing in confined impinging jets. AIChE J. *49*, 2264–2282.
17. Saad, W.S., and Prud'homme, R.K. (2016). Principles of nanoparticle formation by flash nanoprecipitation. Nano Today *11*, 212–227.
18. Pagels, R.F., Edelstein, J., Tang, C., and Prud'homme, R.K. (2018). Controlling and predicting nanoparticle formation by block copolymer directed rapid precipitations. Nano Lett. *18*, 1139–1144.
19. Hu, Y., He, Z., Hao, Y., Gong, L., Pang, M., Howard, G.P., Ahn, H.-H., Brummet, M., Chen, K., Liu, H.-W., et al. (2019). Kinetic control in assembly of plasmid DNA/polycation complex nanoparticles. ACS Nano *13*, 10161–10178.
20. Santos, J.L., Ren, Y., Vandermark, J., Archang, M.M., Williford, J.-M., Liu, H.-W., Lee, J., Wang, T.-H., and Mao, H.-Q. (2016). Continuous production of discrete plasmid DNA-polycation nanoparticles using flash nanocomplexation. Small *12*, 6214–6222.
21. He, Z., Santos, J.L., Tian, H., Huang, H., Hu, Y., Liu, L., Leong, K.W., Chen, Y., and Mao, H.-Q. (2017). Scalable fabrication of size-controlled chitosan nanoparticles for oral delivery of insulin. Biomaterials *130*, 28–41.
22. Hanwright, P.J., Qiu, C., Rath, J., Zhou, Y., von Guionneau, N., Sarhane, K.A., Harris, T.G.W., Howard, G.P., Malapati, H., Lan, M.J., et al. (2022). Sustained IGF-1 delivery ameliorates effects of chronic denervation and improves functional recovery after peripheral nerve injury and repair. Biomaterials *280*, 121244.
23. Li, L., Zhang, Y., Zhou, Y., Hu, H., Hu, Y., Georgiades, C., Mao, H.-Q., and Selaru, F.M. (2022). Quaternary nanoparticles enable sustained release of bortezomib for hepatocellular carcinoma. Hepatology *76*, 1660–1672.
24. Zhu, Y., Shen, R., Vuong, I., Reynolds, R.A., Shears, M.J., Yao, Z.-C., Hu, Y., Cho, W.J., Kong, J., Reddy, S.K., et al. (2022). Multi-step screening of DNA/lipid nanoparticles and co-delivery with siRNA to enhance and prolong gene expression. Nat. Commun. *13*, 4282.
25. Hu, H., Yang, C., Li, M., Shao, D., Mao, H.-Q., and Leong, K.W. (2021). Flash technology-based self-assembly in nanoformulation: Fabrication to biomedical applications. Mater. Today *42*, 99–116.
26. Li, S., Hu, Y., Li, A., Lin, J., Hsieh, K., Schneiderman, Z., Zhang, P., Zhu, Y., Qiu, C., Kokkoli, E., et al. (2022). Payload distribution and capacity of mRNA lipid nanoparticles. Nat. Commun. *13*, 5561.
27. Beh, C.W., Pan, D., Lee, J., Jiang, X., Liu, K.J., Mao, H.-Q., and Wang, T.-H. (2014). Direct interrogation of DNA content distribution in nanoparticles by a novel microfluidics-based single-particle analysis. Nano Lett. *14*, 4729–4735.
28. Liu, K.J., and Wang, T.-H. (2008). Cylindrical illumination confocal spectroscopy: Rectifying the limitations of confocal single molecule spectroscopy through one-dimensional beam shaping. Biophys. J. *95*, 2964–2975.
29. Rui, Y., Wilson, D.R., Tzeng, S.Y., Yamagata, H.M., Sudhakar, D., Conge, M., Berlinicke, C.A., Zack, D.J., Tuesca, A., and Green, J.J. (2022). High-throughput and high-content bioassay enables tuning of polyester nanoparticles for cellular uptake, endosomal escape, and systemic in vivo delivery of mRNA. Sci. Adv. *8*, eabk2855.
30. Kilchrist, K.V., Dimobi, S.C., Jackson, M.A., Evans, B.C., Werfel, T.A., Dailing, E.A., Bedingfield, S.K., Kelly, I.B., and Duvall, C.L. (2019). Gal8 visualization of endosome disruption predicts carrier-mediated biologic drug intracellular bioavailability. ACS Nano *13*, 1136–1152.
31. Bus, T., Traeger, A., and Schubert, U.S. (2018). The great escape: how cationic polyplexes overcome the endosomal barrier. J. Mater. Chem. B *6*, 6904–6918.
32. Hao, Y., Seo, J.-H., Hu, Y., Mao, H.-Q., and Mittal, R. (2020). Flow physics and mixing quality in a confined impinging jet mixer. AIP Adv. *10*, 045105.
33. Kutner, R.H., Zhang, X.-Y., and Reiser, J. (2009). Production, concentration and titration of pseudotyped HIV-1-based lentiviral vectors. Nat. Protoc. *4*, 495–505.

34. Nguyen, T.N.T., Sha, S., Hong, M.S., Maloney, A.J., Barone, P.W., Neufeld, C., Wolfrum, J., Springs, S.L., Sinskey, A.J., and Braatz, R.D. (2021). Mechanistic model for production of recombinant adeno-associated virus via triple transfection of HEK293 cells. Mol. Ther. Methods Clin. Dev. *21*, 642–655.

35. Huang, X., Hartley, A.-V., Yin, Y., Herskowitz, J.H., Lah, J.J., and Ressler, K.J. (2013). AAV2 production with optimized N/P ratio and PEI-mediated transfection results in low toxicity and high titer for in vitro and in vivo applications. J. Virol. Methods *193*, 270–277.

36. Zhao, H., Lee, K.-J., Daris, M., Lin, Y., Wolfe, T., Sheng, J., Plewa, C., Wang, S., and Meisen, W.H. (2020). Creation of a high-yield AAV vector production platform in suspension cells using a design-of-experiment approach. Mol. Ther. Methods Clin. Dev. *18*, 312–320.

37. Wilson, D.R., Routkevitch, D., Rui, Y., Mosenia, A., Wahlin, K.J., Quinones-Hinojosa, A., Zack, D.J., and Green, J.J. (2017). A triple-fluorophore-labeled nucleic acid pH nanosensor to investigate non-viral gene delivery. Mol. Ther. *25*, 1697–1709.

OMTM, Volume *32*

Supplemental information

Liter-scale manufacturing of shelf-stable plasmid DNA/PEI transfection particles for viral vector production

Yizong Hu, Brendan A. Eder, Jinghan Lin, Sixuan Li, Yining Zhu, Tza-Huei Wang, Ting Guo, and Hai-Quan Mao

Supplemental Methods

The fluorescence deconvolution algorithm has been used to analyze the payload in the nanoparticles. In our previous work, the DNA content in DNA/PEI and DNA/PEI-g-PEG nanoparticles (*Nano Letters* 2014, 14 4729-4735) and the mRNA content in lipid nanoparticles (*Nature Communications* 2022, 13, 5561) were analyzed by this technique. The same method was applied to analyze the pDNA payload in the size-controlled pDNA/PEI nanoparticles described in this report. For the nanoparticles with a measured diameter of 50 nm, 150 nm and 200 nm by dynamic light scattering (DLS), 100% (w/w) of the pDNAs were Cy5 labeled; Nanoparticles with 300 nm and 400 nm were loaded with 2% Cy5-pDNA.

For the 100% labeled condition, the pDNA payload was calculated as follows: First the fluorescence burst size distribution of the single molecule pDNA-Cy5 (D_{DNA}) and 100% Cy5 labeled pDNA/PEI nanoparticles (D_{NP}) were obtained by CICS, quantified by single particle fluorescence analysis, and D_{DNA} is normalized by its number of events. Due to the multiplicative nature of the single particle fluorescence, the distribution of the fluorescence is better described by a lognormal than normal distribution, thus quantified by logarithmic binning. The distribution of the single molecule pDNA-Cy5 (D_{DNA}) was used as a basis and a set of basis distribution $D_{DNA,n}\big|_{n=1,2,\ldots,N}$ were generated by multiplying D_{DNA} by a scaling factor n. For each basis distribution, $D_{DNA,n}$, the sum up of the distribution with all the bins is unity. I_B is the number of bins for the distribution:

$$\sum_{i=1}^{I_B} D_{DNA,n}(i) = 1\Bigg|_{n=1,2,\ldots,N}$$

$D_{DNA,n}(i)$ represents the proportion of the distribution in i-th bin of the basis of n pDNAs. I_B is the number of bins for the distribution. The maximum of n, N, is chosen to be a factor (k, typical around 4-6 times) of the ratio of the geometric mean of D_{NP} ($\mu_{D_{NP}}$) to D_{pDNA} ($\mu_{D_{DNA}}$) to ensure N is large enough to cover the whole payload range.

$$N = k\frac{\mu_{D_{NP}}}{\mu_{D_{DNA}}}$$

An estimated pDNA/PEI nanoparticle distribution (${D_{NP}}^*$) was constructed by summing up all the generated basis distributions of n pDNAs multiplied by a weight (w_n) representing the estimation of the number of nanoparticles encapsulating n pDNAs. The w_n is a non-negative integer that is no larger than the total number of pDNA/PEI nanoparticle events (N_{NP}).

$${D_{NP}}^* = \sum_{n=1}^{N} w_n \times D_{DNA,n}$$

$$w_n \in [0, N_{NP}], n = 1,2, \dots, N$$

The fitted nanoparticle distribution (${D_{NP}}^*$) was compared to that of the true distribution (D_{NP}) by calculating the difference based on each bin in the distribution. For each bin, we have the estimated number of the events in each bin given by

$$N(i)^* = D_{NP}(i)^* = \sum_{n=1}^{N} w_n \times D_{DNA,n}(i)$$

Then the total number of estimated events N^* is

$$N^* = \sum_{i=1}^{I_B} N(i)^* = \sum_{i=1}^{I_B} \sum_{n=1}^{N} w_n \times D_{DNA,n}(i) = \sum_{n=1}^{N} w_n$$

The chi square (χ^2) is calculated as the difference between the estimation and the true distribution, plus the difference between the estimated total number of events and the true value, as shown below:

$$\chi^2 = \sum_{i=1}^{I_b} \frac{(N(i) - N(i)^*)^2}{N(i)} + \alpha(N_{NP} - N^*)^2$$

A penalty factor (α) was chosen to be 0.1 to ensure N^* is < 1% off from N_{NP}.

The best fit was optimized by minimizing χ^2 using a simulated annealing algorithm in MATLAB, which gave the best estimate of the number of nanoparticles encapsulating the corresponding number of pDNAs per particle, w_n. The frequency f_n of the nanoparticles encapsulating n copies of pDNA was calculated as

$$f_n = \left.\frac{w_n}{N_{NP}}\right|_{n=1,2,\dots,N}$$

The average payload of the whole population, μ, was calculated as

$$\mu = \sum_{n=1}^{N} n \times f_n$$

For the 100% pDNA-Cy5 labeled condition (i.e., 50 nm, 150 nm, and 200 nm nanoparticles), the payload distributions and average payload were obtained as described above.

Modifications were made to compensate for the nanoparticles with 2% pDNA-Cy5 labeling (i.e., 300 nm, and 400 nm nanoparticles). First, the payload distribution at 2% labeled condition, $f_{n,2\%}$, with payload number range from 1 to $N_{2\%}$, was calculated as described above. Since only 2% pDNAs were Cy5 labeled,

a significant number of nanoparticles may not have a fluorescent label. The number of the Cy5 labeled pDNAs in the particles, N, follows a binomial distribution, B:

$$P(N = x) \sim B(x, n, p)$$

Where n is the detected number of payload, p is the labeling ratio ($p = 0.02$).

The portion of the unlabeled nanoparticles was given by:

$$P(N = 0) = \sum_{n=1}^{N_{2\%}} B(0, n, p) \times f_{n,2\%}$$

An overestimation factor K_{oest} due to neglecting the portion of nanoparticle that encapsulated no Cy5-labeled pDNA (n = 0) in the payload quantification was calculated as:

$$K_{oest} = \frac{1}{1 - P(N = 0)}$$

And to include the nonfluorescent portion in the payload distribution, $f_{n,2\%}$ was corrected given by

$$f_{n,2\%,corr} = \frac{[P(N = 0), f_{n,2\%}]}{sum([P(N = 0), f_{n,2\%}])}$$

Next, to back calculate the complete payload distribution from the 2% labeled results, a matrix mapping the binomial distribution of the 100% to 2% probability, M was constructed with the entry in n-th row and m-th column:

$$M_{n,m} = B(x, n, p)\big|_{n=1,2,\ldots,N_{100\%},\ \ m=1,2,\ldots,N_{2\%}+1}$$

Where $N_{100\%}$ is the largest payload number in the complete payload distribution, and $N_{2\%}$ is the highest payload in the payload distribution at 2% labeled condition, $f_{n,2\%}$. The 100% probability was obtained by dividing the corrected 2% payload distribution by the binomial probability matrix:

$$f_{n,100\%} = f_{n,2\%,corr}/M$$

And the average payload of the whole population, μ, was calculated as

$$\mu = \frac{\sum_{n=1}^{N_{100\%}} n \times f_{n,100\%}}{K_{oest}}$$

Supplemental Figures

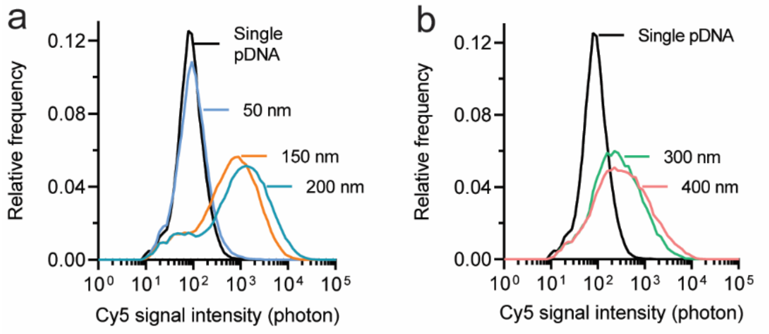

Figure S1. The distribution of Cy5 signal intensity from pDNA/PEI nanoparticle events detected by cylindrical illumination confocal spectroscopy (CICS) single-nanoparticle characterization technique.

(a) For pDNA/PEI nanoparticles loaded with 100% Cy5-pDNA at a size of 50, 150, and 200 nm, referencing to the signal intensity distribution of individual free Cy5-pDNAs; **(b)** For pDNA/PEI nanoparticles loaded with 2% Cy5-pDNA and 98% non-labeled pDNA at a size of 300 and 400 nm.

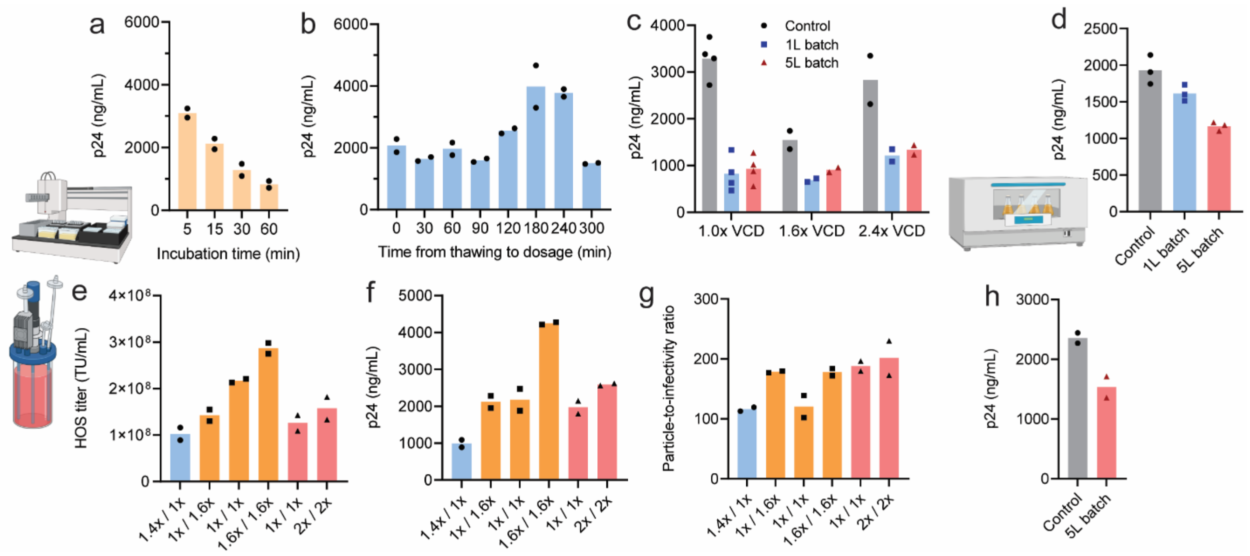

Figure S2. Additional data from lentiviral vector production using stable pDNA/PEI nanoparticles (NPs).

Additional data from lentiviral vector production is shown for **(a–c)** Ambr 15 culture system; **(d)** 100-mL shake flask system; and **(e–h)** 2-L in-house developed single-use bioreactors.

(a) p24 capsid protein quantifications from harvested LVVs produced by the conventional pDNA-PEI mixing method (control, main text **Figure 1a**), and the time on x axis indicates the time between mixing pDNA with PEI and dosing; **(b)** p24 capsid protein quantifications from harvest LVVs produced by stable NPs (the 1-L lot), and the time on x axis indicates the time between complete thawing and dosing under ambient temperature. **(c)** p24 capsid protein quantifications from harvested LVVs comparing the control with 1-L and 5-L lot NPs. 1x VCD represents the standard viable cell density at 24 h prior to NP dosing, while the dose was proportional to the VCD level. **(d)** p24 capsid protein quantifications from harvested LVVs comparing the control with 1-L and 5-L lot NPs in the 100-mL shake flask system with 1x VCD and 1x dose. **(e–g)** The 1-L lot NPs were used. The quantity and quality of LVVs produced in the experiments that varied dose and VCD are reported, and the x axis reads VCD level / NP dosing level. For example, 1.4x / 1x reads 1.4x VCD and 1x NP dose, in which 1x dose is the standard condition of 1 μg total pDNA/mL in culture. The experiments were carried out in three independent batches of bioreactor runs, and the columns with the same color (either blue, orange, or red) were from parallel experiments within the same batch of bioreactor run. **(h)** p24 capsid protein quantifications from harvested LVVs comparing the control with 5-L lot NPs in the 2-L bioreactor with 1x VCD and 1x dose.

Supplemental Videos

In all supplemental videos, the red color indicates Gal8-mRuby3, while the blue color indicates Cy5-tagged pDNA.

Video S1. The full recorded video of B16F10-Gal8-mRuby3 cells treated by 400-nm NPs, with all Z planes stacked to give a top-down view of a 3-D perspective.

Video S2. The full recorded video of B16F10-Gal8-mRuby3 cells treated by 50-nm NPs, at a single focal plane of Z = 10.

Video S3. The full recorded video of B16F10-Gal8-mRuby3 cells treated by 400-nm NPs, at a single focal plane of Z = 10.
